# Cross-sectional analyses between the dietary index for gut microbiota and Parkinson’s disease in the middle-aged and elderly population

**DOI:** 10.1186/s12937-025-01206-5

**Published:** 2025-09-30

**Authors:** Yiming Zhan, Yuhang Liu, Huxingzi Liu, Siyao Gao, Jialing Tang

**Affiliations:** 1 Department of Physical Education, Central South University, No.932 South Lushan Road, Yuelu District, Changsha, 410083 P. R. China; 2 School of Physical Education and Sports, Central China Normal University, Wuhan, 430079 P. R. China

**Keywords:** Parkinson's disease, Dietary index for gut microbiota, Cross-sectional study, Neurodegenerative disease, DI-GM

## Abstract

**Background:**

The evidence linking dietary indices to Parkinson’s disease (PD) risk remains limited. This study investigated the association between the Dietary Index for Gut Microbiota (DI-GM) and PD risk in middle-aged and elderly populations.

**Methods:**

Cross-sectional data were obtained from the 2007–2020 cycles of the National Health and Nutrition Examination Survey (NHANES), including 17,373 participants aged 40 years or older. PD was defined by antiparkinsonian medication use. DI-GM scores were calculated based on 14 dietary components. Multiple logistic regression and a restricted cubic spline model (RCS) were used to test the relationship between DI-GM and PD. Subgroup and sensitivity analyses were conducted to evaluate the robustness of the results.

**Results:**

Among 17,373 participants, those with PD (*n* = 232) had lower DI-GM scores compared to non-PD individuals. Higher DI-GM scores (≥ 6) were associated with a 49% reduction in PD prevalence. RCS analysis showed a non-linear relationship between DI-GM and PD (*P* < 0.05). Subgroup and sensitivity analyses confirmed stable associations across demographic groups and after adjusting for comorbidities.

**Conclusions:**

A gut microbiota-friendly diet, as measured by DI-GM, is associated with reduced PD risk, especially in the higher DI-GM score. This finding may inform future dietary guidelines for the prevention of PD.

**Supplementary Information:**

The online version contains supplementary material available at 10.1186/s12937-025-01206-5.

## Highlights

• DI-GM score ≥ 6 linked to 49% lower PD risk.

• First study showing DI-GM’s nonlinear inverse association with PD.

• Robust diet-PD link confirmed across diverse demographic subgroups.

• Restricted cubic spline models reveal a nonlinear DI-GM-PD relationship.

• Large NHANES sample (*n* = 17,373) supports diet-microbiota-PD findings.

**Supplementary Information:**

The online version contains supplementary material available at 10.1186/s12937-025-01206-5.

## Introduction

Parkinson’s disease (PD) is a progressive neurological disorder characterized by tremor, rigidity, and bradykinesia, primarily associated with the progressive loss of dopaminergic neurons in the substantia nigra pars compacta and other brain structures [1]. As the second most common neurodegenerative disorder after Alzheimer’s disease, PD poses a significant public health challenge globally. Recent epidemiological projections suggest a significant 150% increase in the prevalence of PD by 2040, largely driven by population aging. Within the United States, the annual economic burden of PD has been quantified at approximately $52 billion, with inpatient care services accounting for 32% and pharmacological interventions representing 21% of the total healthcare expenditures associated with this neurodegenerative disorder [2]. The disease not only impacts patients’ quality of life but also places a heavy burden on caregivers and healthcare systems, particularly in low and middle-income countries where access to advanced treatments is limited [3]. The global impact of PD is particularly pronounced among the elderly population, affecting approximately 1.5% of individuals aged 65 and above, and 3.0% of those aged 80 and above [4]. With the ongoing expansion of the global aging demographic, the epidemiological impact of PD has intensified, positioning it as an increasingly significant public health challenge worldwide [5].

Accumulating evidence indicates that alterations in gut microbiota composition, characterized by dysbiosis, may significantly contribute to the pathogenesis of PD, notably through the reduced synthesis of neuroprotective metabolites such as butyrate, with concentrations typically below 0.5mM in PD patients compared to levels exceeding 1.2mM in healthy controls [6]. Dietary patterns, as a modifiable lifestyle factor, have been shown to significantly influence gut microbiota composition and function, thereby potentially affecting neurodegenerative processes [7]. An inappropriate dietary pattern is closely related to increased inflammatory factors in PD, often caused by oxidative stress and chronic neuroinflammation. Emerging research highlights the bidirectional communication between the gut and the brain (the gut-brain axis), where microbial metabolites regulate neuroinflammation, blood-brain barrier integrity, and α-synuclein aggregation—a pathological hallmark of PD [8, 9]. The Dietary Index for Gut Microbiota (DI-GM), a novel scoring system developed by Kase et al., provides a comprehensive assessment of both beneficial and detrimental dietary components that influence the composition of gut microbiota. Higher DI-GM scores indicate a diet more likely to promote gut microbiota diversity, while lower scores suggest a diet associated with dysbiosis [10]. This index integrates 14 dietary components, including fiber-rich foods (e.g., whole grains, legumes) and fermented products (e.g., yogurt), which enhance microbial production of short-chain fatty acids (SCFAs), alongside pro-inflammatory components such as red meat and refined grains [11, 12].

Although previous studies on DI-GM have demonstrated significant inverse associations with conditions such as depression and stroke—both of which share inflammatory and metabolic pathways with PD—these studies have not specifically explored its relevance to PD [13, 14]. Early epidemiologic studies of diet and PD have focused on isolated nutrients (e.g., caffeine, polyphenols) or broad dietary patterns, such as the Mediterranean diet [15, 16]. However, few have used microbiota-centered indices such as the DI-GM. To fill this gap, we aimed to assess the association between DI-GM and PD risk using data from the NHANES 2007–2020 cycle. Based on new evidence linking diet, gut microbiota, and neurological health, we hypothesized that higher DI-GM scores, reflecting a more diet-friendly microbiota, may result in a lower risk of PD in middle-aged and older adults.

## Materials and methods

### Study design and population

**Fig. 1:**
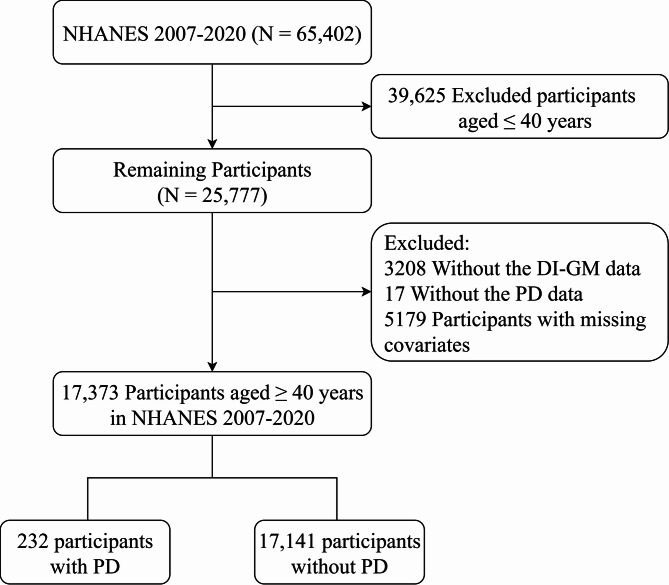
The flow chart of selection of included studies

The data utilized in this study are derived from the NHANES spanning the years 2007 to 2020. NHANES represents one of the most comprehensive health-related initiatives conducted by the National Center for Health Statistics (NCHS) in collaboration with the Centers for Disease Control and Prevention, consistently providing data for public access [17]. This study adheres to the principles of the Declaration of Helsinki. The survey comprised a cross-sectional assessment aimed at generating nationally representative data on the civilian, noninstitutionalized population of the United States. Among the sample of 65,402 adults, those who did not provide DI-GM or other variables were excluded (Fig. 1). The data collection protocol received approval from the Ethical Review Board of the NCHS, and all participants provided informed consent before their interviews and examinations. Consequently, this investigation did not require any further institutional review board approval or informed consent. Access to all NHANES data is freely available on the following websites: https://wwwn.cdc.gov/nchs/nhanes/Default.aspx.

### Assessment of PD

The diagnosis of PD in this study was determined based on the use of antiparkinsonian medications, as identified in the NHANES Prescription Medications document. Participants were classified as having PD if their prescribed medications were categorized under the “Second Level Category Name” as “ANTIPARKINSON AGENTS” [16, 18]. This classification relied on participants’ self-reported use of prescribed medications, consistent with the methodology employed in prior research. Individuals who did not report the use of antiparkinsonian medications were classified as non-PD participants. Although this approach may lead to misclassification of undiagnosed or untreated PD cases, it has been used in previous NHANES-based epidemiologic studies and remains the most feasible approach based on the available data [19].

### Assessment of DI-GM

Based on a scale developed by Kase et al., the DI-GM consists of 14 items, 10 food items considered beneficial to gut microbiota: avocado, broccoli, chickpeas, coffee, cranberries, fermented dairy, fiber, green tea, soybeans, and whole grains. The 4 harmful items include red meat, processed meat, refined grains, and high-fat diets. For each beneficial item, participants were given a score of 1 if their intake was above the gender-specific median, and a score of 0 otherwise. For the harmful items, a score of 1 was given if intake was below the gender-specific median or if no intake occurred; otherwise, a score of 0 was given. The total DI-GM score ranged from 0 to 13 (As NHANES does not record the specific types of tea consumed, data were not available.), with higher scores indicating that the diet was more favorable to the microbiota (Supplementary Table 1) [10, 13]. The DI-GM was calculated based on a single 24-hour dietary recall, which is the only dietary intake data available for most participants during the included NHANES cycles.

### Covariates

In this study, potential confounding variables were carefully selected and adjusted based on existing literature and clinical relevance. These variables included age, sex, race/ethnicity, marital status, educational attainment, poverty-to-income ratio (PIR), alcohol consumption, smoking status, body mass index (BMI), sedentary behavior (SB) time, and sleep duration. For logistic regression analysis, age was treated as a continuous variable, while for participant characterization and subgroup analyses, it was stratified into two groups: 40–59 years and ≥ 60 years. Race/ethnicity was categorized as Non-Hispanic White, Non-Hispanic Black, Mexican American, and other races. Marital status was classified into three groups: married or living with a partner, never married, and widowed/divorced/separated. Educational attainment was divided into three levels: below high school, high school, and some college or higher. PIR was categorized into three tiers: ≤1.30, 1.30–3.50, and > 3.50. Smoking status was determined based on responses to two questions: “Have you smoked at least 100 cigarettes in your lifetime?” and “Do you currently smoke?” Participants were classified as never smokers, former smokers, or current smokers. Alcohol consumption was self-reported and categorized into four groups: never drinkers (fewer than 12 drinks in a lifetime), former drinkers (at least 12 drinks in a year but not in the past year), mild drinkers (≤ 1 drink per day for females and ≤ 2 drinks per day for males), and heavy drinkers (≥ 3 drinks per day for males and ≥ 4 drinks per day for females) [20]. These adjustments were implemented to minimize confounding effects and ensure a robust analysis of the associations under investigation.

### Statistical analysis

All statistical analyses were conducted by the NHANES sampling design, incorporating appropriate weighting to ensure the representativeness of the results. Continuous variables were expressed as means with standard errors (SE) based on their distributions and were compared using t-tests. Categorical variables were expressed as numerical counts and percentage frequencies (%) and analyzed using weighted Chi-square tests. To examine the relationship between DI-GM and PD, multivariate logistic regression analysis was employed. Odds ratios (OR) and their corresponding 95% confidence intervals (CI) were calculated to assess the associations. Weighted logistic regression models were used to adjust for various demographic and health-related covariates. Model 1 was the crude model, with no adjustments for covariates. Model 2 was adjusted for age, sex, race/ethnicity, marital status, education level, and PIR. Model 3 included the same variables as Model 2, with additional adjustments for BMI, alcohol consumption, smoking status, sedentary behavior (SB) time, and sleep duration. Additionally, restricted cubic splines (RCS) were used in weighted logistic regression models to assess the dose-response relationship between DI-GM and PD, allowing for the visualization of potential non-linear associations. Subgroup analyses were performed to explore potential variations in the effect of DI-GM on PD across different demographic groups. To assess the robustness of the results, sensitivity analyses were conducted by incorporating additional covariates such as diabetes mellitus, hypertension, and cardiovascular disease. All statistical analyses were performed using R software (version 4.4.1) from the R Project for Statistical Computing. Statistical tests were two-sided, with a significance level set at *P* < 0.05.

## Results

### Baseline characteristics of participants

Following the exclusion of participants with missing covariate data, the final analytical sample comprised 17,373 individuals (mean age = 57.59 ± 0.17 years; 47.43% male), among whom 232 (1.29%) were diagnosed with PD (Table 1). Participants with PD (mean age = 60.88 ± 1.12, mean DI-GM = 4.40 ± 0.14) were significantly older than non-PD individuals and had lower DI-GM scores (mean age = 57.55 ± 0.17; *P* < 0.05, mean DI-GM = 4.86 ± 0.03; *P* < 0.05). Additionally, 62.12% of the participants were men; 81.03% were of Non-Hispanic White, and those with lower socioeconomic status. Significant differences were observed in lifestyle and health-related factors. Participants with PD had a higher prevalence of current smoking, cardiovascular disease, hypertension, and stroke (All *P* < 0.05). Supplementary Table 2 provides baseline data based on the DI-GM, categorized by different score ranges: 0–3, 4, 5, and ≥ 6. Additionally, we present baseline data categorized by gender and age groups (40–59 years and over 60 years) in Supplementary Tables 3 and Supplementary Table 4, respectively.

**Table 1:** Sample characteristics, by the PD, NHANES 2007–2020 (*n* = 17,373)

Characteristics	Total	Without PD	PD	*P*-value^a^
Age, years	57.59 ± 0.17	57.55 ± 0.17	60.88 ± 1.12	**< 0.05**
BMI	29.41 ± 0.09	29.41 ± 0.09	29.83 ± 0.59	0.47
DI-GM	4.86 ± 0.03	4.86 ± 0.03	4.40 ± 0.14	**< 0.05**
Beneficial to gut microbiota	2.25 ± 0.02	2.25 ± 0.02	1.88 ± 0.10	**< 0.001**
Unfavorable to gut microbiota	2.61 ± 0.01	2.61 ± 0.01	2.52 ± 0.09	0.34
SB time	375.40 ± 3.05	375.13 ± 3.05	395.55 ± 19.58	0.3
Sleep duration	7.14 ± 0.02	7.14 ± 0.02	7.24 ± 0.15	0.47
Race/ethnicity				**0.05**
Non-Hispanic Black	3769 (9.68)	3738 (9.70)	31 (7.61)	
Non-Hispanic White	7889 (73.36)	7738 (73.26)	151 (81.03)	
Mexican American	2307 (5.94)	2285 (5.97)	22 (3.80)	
Other races	3408 (11.02)	3380 (11.06)	28 (7.56)	
Sex				**< 0.05**
Female	8774 (52.57)	8647 (52.44)	127 (62.12)	
Male	8599 (47.43)	8494 (47.56)	105 (37.88)	
Education level				0.32
< high school	4218 (15.10)	4157 (15.05)	61 (18.62)	
High school	3970 (23.41)	3916 (23.37)	54 (26.40)	
College or above	9185 (61.50)	9068 (61.59)	117 (54.97)	
Marital level				0.19
Married/Living with partner	10,882 (67.99)	10,745 (68.08)	137 (61.77)	
Widowed/Divorced/Separated	5051 (24.92)	4985 (24.88)	66 (27.43)	
Never married	1440 (7.09)	1411 (7.04)	29 (10.80)	
PIR				**< 0.05**
< 1.3	4836 (17.29)	4757 (17.20)	79 (24.49)	
>3.5	5985 (49.10)	5929 (49.30)	56 (34.08)	
1.3–3.5	6552 (33.60)	6455 (33.50)	97 (41.44)	
Smoking status				**< 0.05**
Never	8979 (51.84)	8861 (51.85)	118 (51.28)	
Former	5212 (30.61)	5148 (30.73)	64 (21.83)	
Now	3182 (17.55)	3132 (17.43)	50 (26.89)	
Alcohol consumption				**< 0.05**
Former	3174 (15.86)	3108 (15.74)	66 (25.15)	
Heavy	2591 (15.35)	2564 (15.35)	27 (14.84)	
Never	2408 (10.33)	2375 (10.34)	33 (9.80)	
Moderate	2515 (16.95)	2496 (17.06)	19 (8.97)	
Mild	6685 (41.50)	6598 (41.51)	87 (41.25)	
DI-GM group				**< 0.05**
0–3	4010 (20.71)	3938 (20.56)	72 (32.19)	
4	4013 (21.24)	3965 (21.28)	48 (18.18)	
5	4078 (24.03)	4027 (24.03)	51 (24.17)	
≥ 6	5272 (34.02)	5211 (34.13)	61 (25.46)	
CVD				**< 0.001**
No	14,754 (87.62)	14,596 (87.79)	158 (75.33)	
Yes	2619 (12.38)	2545 (12.21)	74 (24.67)	
DM				**< 0.05**
DM	4450 (19.86)	4369 (19.79)	81 (24.81)	
IFG	997 (5.52)	982 (5.46)	15 (9.96)	
IGT	713 (3.87)	701 (3.87)	12 (4.21)	
No	11,213 (70.75)	11,089 (70.88)	124 (61.03)	
Hypertension				**< 0.001**
No	7660 (49.68)	7587 (49.90)	73 (33.27)	
Yes	9713 (50.32)	9554 (50.10)	159 (66.73)	
Stroke				**< 0.001**
No	16,427 (95.77)	16,227 (95.87)	200 (87.88)	
Yes	946 (4.23)	914 (4.13)	32 (12.12)	

Continuous variables are presented as mean ± SE, and categorical variables are presented as *n* (weighted%)

^a^*P*-values were assessed by T-test (continuous variables) or by Chi-square test (categorical variables). *P*-values shown in bold were statistically significant

*Abbreviations* *BMI* Body mass index, *CVD* Cardiovascular disease, *DM* Diabetes mellitus, *DI-GM* Dietary index for gut microbiota, *SB* Sedentary behavior, *NHANES* National Health and Nutrition Examination Survey, *PIR* Poverty income ratio, *SE* Standard error, *PD* Parkinson’s disease, *IFG* Impaired fasting glucose, *IGT* Impaired glucose tolerance

### Association between DI-GM and PD

**Fig. 2:**
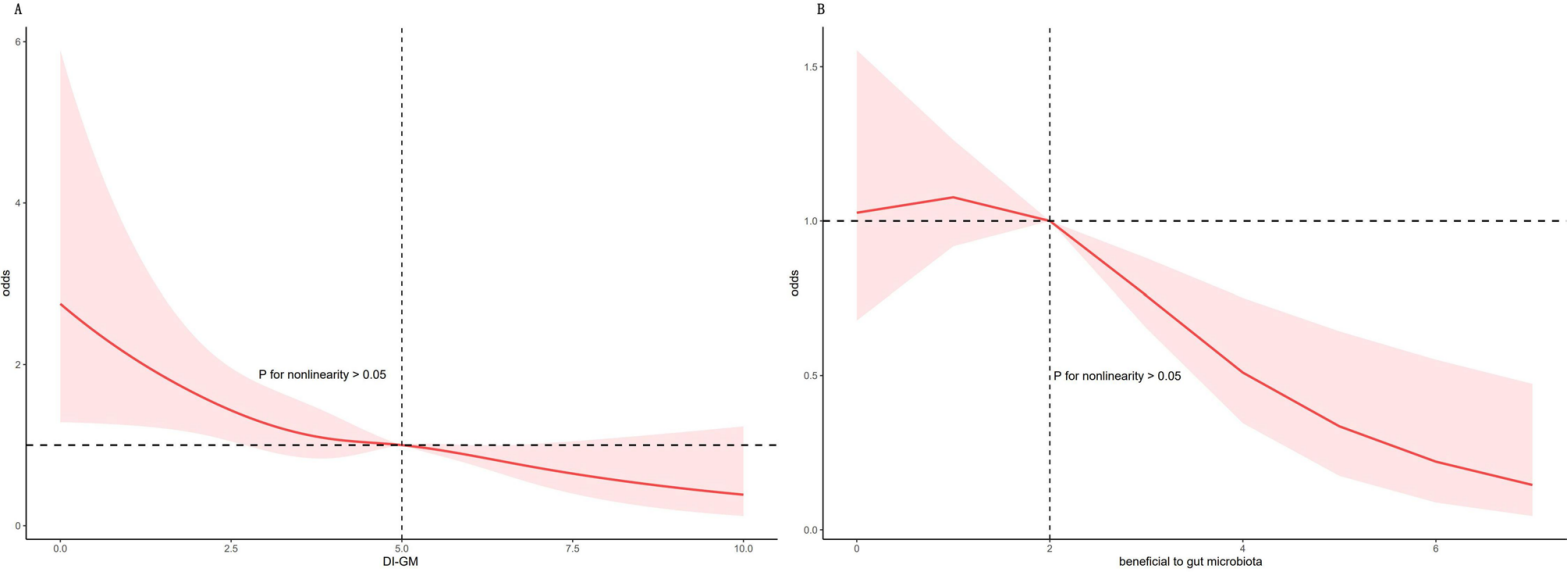
Dose-response relationship between DI-GM (**A**), beneficial to gut microbiota (**B**), and PD, NHANES 2007-2020 (*n* = 13, 796). The model adjusted for age, sex, race/ethnicity, education level, marital status, PIR, smoking status, sleep duration, SB time, BMI and alcohol consumption. Abbreviation: NHANES, National Health and Nutrition Examination Survey; PIR, Poverty income ratio; BMI, Body mass index; DI-GM, Dietary index for gut microbiota; SB, Sedentary behavior

As shown in Table 2, the association between DI-GM and PD was significant in both Model 1 and Model 2 (OR = 0.85, 95% CI = 0.76, 0.94; OR = 0.85, 95% CI = 0.76, 0.95). After categorizing DI-GM into groups, a significant inverse correlation was observed between the DI-GM scores (≥ 6) and the prevalence of PD (OR = 0.49, 95% CI = 0.30, 0.78; OR = 0.50, 95% CI = 0.31, 0.82). Additionally, the beneficial components of DI-GM showed a significant reduction in PD risk in both adjusted model 1 and model 2 (OR = 0.83, 95% CI = 0.73, 0.93; OR = 0.83, 95% CI = 0.73, 0.94). The trend analysis revealed that as DI-GM scores increased, the risk of PD decreased significantly (*P* for trend < 0.05). RCS analysis showed a non-linear relationship between DI-GM and PD (*P* < 0.05), and the beneficial components of DI-GM (*P* > 0.05) also showed a non-linear correlation with PD (Fig. 2).

**Table 2:** Association between DI-GM and PD of the NHANES 2007–2020 participants

	Crude model		Model 1		Model 2	
	COR(95%CI)	*P*-value	AOR(95%CI)	*P*-value	AOR(95%CI)	*P*-value
DI-GM	0.84(0.76,0.94)	**< 0.01**	0.85(0.76, 0.94)	**< 0.01**	0.85(0.76, 0.95)	**0.01**
DI-GM group						
0–3	Reference		Reference		Reference	
4	0.55(0.33,0.91)	**< 0.05**	0.56(0.33, 0.95)	**< 0.05**	0.58(0.34, 0.98)	**< 0.05**
5	0.64(0.37,1.10)	0.11	0.65(0.37, 1.16)	0.14	0.65(0.37, 1.14)	0.13
≥ 6	0.48(0.30,0.75)	**< 0.01**	0.49(0.30, 0.78)	**< 0.01**	0.50(0.31, 0.82)	**0.01**
*p* for trend		**0.01**		**0.01**		**< 0.05**
Unfavorable to gut microbiota	0.93(0.79,1.08)	0.33	0.90(0.77, 1.05)	0.18	0.91(0.78, 1.07)	0.24
Beneficial to gut microbiota	0.81(0.72,0.91)	**< 0.01**	0.83(0.73, 0.93)	**< 0.01**	0.83(0.73, 0.94)	**< 0.01**

The crude model was unadjusted. Model 1 was adjusted for age, sex, race/ethnicity, education level, marital status, BMI, and PIR. Model 2 was additionally adjusted for smoking status, sleep duration, SB time, and alcohol consumption. The results of COR (95% CI), AOR (95% CI), and *P*-value shown in bold were statistically significant. *P*-value < 0.05 or *P*-value < 0.01

*Abbreviations* *AOR* Adjusted odds ratio, *CI* Confidence interval, *DI-GM* Dietary index for gut microbiota, *COR* Crude odds ratio, *NHANES* National Health and Nutrition Examination Survey, *PIR* Poverty income ratio, *SB* Sedentary behavior, *BMI* body mass index, *PD* Parkinson’s disease

### Subgroup analyses and sensitivity analyses

The results of subgroup analyses are detailed in Table 3. Following adjustment for covariates, the magnitude of associations within each subgroup exhibited minimal variation. The association between DI-GM and reduced PD risk was consistently observed across all predefined subgroups, with no significant interaction effects detected (all interaction *P* > 0.05).

**Table 3:** Subgroup of the association of DI-GM with PD, NHANES 2007–2020 (*n* = 7255)

Subgroup	AOR(95% CI)	*P*-value	*P*-interaction
Sex			0.39
Female	0.82 (0.72,0.94)	0.004	
Male	0.91 (0.73,1.12)	0.36	
Race/ethnicity			0.12
Non-Hispanic Black	1.14 (0.87, 1.50)	0.34	
Non-Hispanic White	0.83 (0.73,0.94)	0.005	
Mexican American	0.80 (0.58, 1.10)	0.16	
Other races	0.79 (0.67,0.93)	0.005	
Education level			0.49
< high school	0.93 (0.79,1.09)	0.35	
High school	0.89 (0.73, 1.08)	0.22	
College or above	0.81 (0.69,0.95)	0.01	
Smoking status			0.5
Never	0.80 (0.69,0.93)	0.01	
Former	0.88 (0.72,1.08)	0.21	
Now	0.93 (0.73,1.18)	0.56	
Marital status			0.47
Married/Living with partner	0.89 (0.77, 1.03)	0.12	
Widowed/Divorced/Separated	0.76 (0.60,0.96)	0.02	
Never married	0.85 (0.61,1.18)	0.32	
PIR			0.83
1.3–3.5	0.83 (0.72,0.95)	0.01	
>3.5	0.84 (0.68,1.05)	0.12	
< 1.3	0.90 (0.74,1.10)	0.30	
Age			0.34
60-	0.88 (0.76,1.02)	0.08	
40–59	0.81 (0.67,0.98)	0.03	

The multivariable logistic regression model was adjusted for gender, race/ethnicity, education level, marital status, and PIR. The results of AOR (95% CI), P-interaction, and *P*-value shown in bold were statistically significant. *P*-value < 0.05

*Abbreviations* *DI-GM* Dietary index for gut microbiota, *NHANES* National Health and Nutrition Examination Survey, *PIR* Poverty income ratio, *PD* Parkinson’s disease

Sensitivity analyses yielded results that were closely aligned with the primary findings. The inverse relationship between DI-GM and PD risk retained statistical significance (*P* < 0.05) after additional adjustments for metabolic and cardiovascular comorbidities, including diabetes, hypertension, and stroke (Supplementary Table 5).

## Discussion

This cross-sectional study revealed a significant inverse association between the DI-GM and PD risk, with participants scoring ≥ 6 on the DI-GM exhibiting a 49% reduction in PD prevalence compared to those with lower scores. These associations persisted across multiple models, adjusting for confounders. Subgroup analyses and sensitivity analyses confirmed their stability across diverse demographic groups.

Emerging evidence supports a role for the gut-brain axis in the pathogenesis of PD, with diet being a key regulator of gut microbiota composition and function. This axis may be the key mechanism explaining our findings. Microbe-supportive dietary components, such as fiber and polyphenols, are central to this gut-brain communication. They promote the production of SCFAs such as butyrate, which crosses the blood-brain barrier, exerts anti-inflammatory and neuroprotective effects, reduces oxidative stress, and supports neuronal survival [6, 12, 21]. Our findings demonstrate a significant inverse correlation between the DI-GM and PD, corroborating existing epidemiological evidence associating specific dietary patterns with neurodegenerative disease pathogenesis. This is supported by studies showing that higher dietary fiber intake—a key DI-GM component—is associated with reduced PD risk, likely mediated by gut microbiota-derived short-chain fatty acids (SCFA) [22]. Furthermore, a meta-analysis by Mischley et al. highlighted the protective role of polyphenol-rich diets, including coffee and green tea (both DI-GM components), in lowering PD incidence, further supporting the importance of microbiota-friendly dietary components [23, 24]. Consumption of more fiber- and polyphenol-rich foods major component of DI-GM was associated with reduced neuroinflammation and increased production of neuroprotective metabolites such as short-chain fatty acids [25, 26]. In contrast, diets high in red and processed meats are associated with systemic inflammation and increased intestinal permeability, which may exacerbate aggregation of α-synuclein and loss of dopaminergic neurons [8]. These mechanisms provide biological plausibility for our findings, suggesting that higher DI-GM scores may be associated with a reduced risk of PD through modulation of the gut-brain axis.

Subgroup and sensitivity analyses further validated the study findings. An inverse association between the DI-GM dietary pattern and reduced PD risk remained consistent across demographic subgroups, with no significant interaction effects detected. Sensitivity analyses adjusting for comorbidities, including diabetes, hypertension, and cardiovascular disease, yielded results nearly identical to the primary analysis, confirming the independence of this association from underlying health conditions. These results align with prior evidence, such as Chen et al.’s study, which reported no significant interaction between dairy consumption (a covariate adjusted in this analysis) and PD risk in stratified analyses, reinforcing the generalizability of dietary recommendations [27].

This study benefits from several strengths, including the use of a nationally representative NHANES sample, standardized DI-GM scoring based on rigorously defined dietary components, and comprehensive adjustment for sociodemographic and clinical confounders. The incorporation of sensitivity and subgroup analyses enhances the reliability and generalizability of the findings. However, limitations must be acknowledged. Firstly, individuals with early-stage PD may unconsciously adopt healthier diets due to gastrointestinal symptoms or olfactory dysfunction, potentially biasing the observed associations. And then although we observed a significant association between DI-GM and PD, we cannot infer causality from this cross-sectional design. In addition, in our study, the diagnosis of PD was based on self-reported use of anti-Parkinson’s disease medications, which may lead to misclassification. Patients with early, undiagnosed, or untreated PD may be misdiagnosed as non-Parkinson’s disease patients. This may reduce the sensitivity of identifying true cases of PD, which may weaken the observed correlation. Conversely, some individuals may be treated with anti-Parkinson’s disease medications for non-Parkinson’s disease indications, thereby slightly reducing specificity. Although this approach is consistent with previous NHANES-based studies, future studies incorporating clinical diagnosis or biomarker confirmation will help improve the accuracy of the classification [19]. Moreover, dietary intake data are collected through self-reported 24-hour recall, which may introduce recall bias. This limitation is particularly important for older adults, who may have difficulty accurately recalling food types or portion sizes, which may result in misclassification of exposure and may affect the accuracy of DI-GM scores. RCS analysis showed a non-linear relationship. Furthermore, while DI-GM provides an indirect dietary proxy for gut microbiota health, our ability to assess specific microbial taxa or functional pathways involved in PD is limited by the lack of direct microbiome data. Future studies should incorporate metagenomic sequencing analyses or metabolomic analyses to validate observed associations and explore mechanistic links between diet, gut microbiota, and neurodegeneration. This integrated approach may provide clearer insights into the gut-brain axis and its role in the pathogenesis of PD.

## Conclusions

This study found a nonlinear inverse relationship between DI-GM and PD through cross-sectional analyses. These findings suggest that microbiota-supportive dietary patterns may be associated with a reduced risk of PD. Future longitudinal and interventional studies are necessary to determine whether microbiota-targeted dietary modifications causally reduce the risk of PD.

## Supplementary Information

Supplementary Material 1: Table S1. Components and scoring criteria of DI-GM in NHANES. Table S2. Sample characteristics, by the DI-GM group, NHANES 2007-2020(*n*=17,373). Table S3. Sample characteristics, by Sex, NHANES 2007-2020(*n*=17,373). Table S4. Sample characteristics, by age, NHANES 2007-2020(*n*=17,373). Table S5. Sensitivity analysis of the association of DI-GM with PD, NHANES 2007-2020 (*n* = 17,373).

## Data Availability

The datasets generated and analyzed during the present study are available from the NHANES databases (Available from https://www.cdc.gov/nchs/nhanes/participant.htm).

## Abbreviations

PD: Parkinson’s Disease
DI-GM: Dietary Index for Gut Microbiota
NHANES: National Health and Nutrition Examination Survey
RCS: Restricted cubic spline
SCFAs: Short-chain fatty acids
NCHS: National Center for Health Statistics
BMI: Body mass index
SB: Sedentary behavior
OR: Odds ratio
CI: Confidence interval
SE: Standard error
