# Supplementary material for: Cross-sectional analyses between the dietary index for gut microbiota and Parkinson’s disease in the middle-aged and elderly population
Source: Nutr J. 2025 Sep 30;24:138. doi: 10.1186/s12937-025-01206-5 (PMC12481752; doi:10.1186/s12937-025-01206-5)
Supplement: Supplementary file 1 — Supplementary Material 1: Table S1. Components and scoring criteria of DI-GM in NHANES. Table S2. Sample characteristics, by the DI-GM group, NHANES 2007-2020(n=17,373). Table S3. Sample characteristics, by Sex, NHANES 2007-2020(n=17,373). Table S4. Sample characteristics, by age, NHANES 2007-2020(n=17,373). Table S5. Sensitivity analysis of the association of DI-GM with PD, NHANES 2007-2020 (n = 17,373). [file 12937_2025_1206_MOESM1_ESM.docx]

**Supplementary Materials**

**Cross-sectional Analyses Between the Dietary Index for Gut Microbiota and Parkinson’s Disease in the Middle-aged and Elderly Population**

## Table S1 Components and scoring criteria of DI-GM in NHANES.

| **Components of DI-GM** | **Food items included in NHANES** | **Scoring criteria** |
| --- | --- | --- |
| **Beneficial to gut microbiota** | Avocados | Score 1 - Consumption≥sex-specific median  Score 0 - Otherwise |
|  | Broccoli |  |
|  | Chickpeas |  |
|  | Coffee |  |
|  | Cranberries |  |
|  | Fermented dairy (including yogurt, cheese, kefir, sour cream, buttermilk) |  |
|  | Fiber |  |
|  | Soybean (including Soy milk, Tofu) |  |
|  | Whole grains |  |
|  | Green tea |  |
| **Unfavorable to gut microbiota** | Refined grains | Score 0 - Consumption≥sex-specific median  Score 1 - Otherwise |
|  | Processed meat |  |
|  | Red meat |  |
|  | High-fat diet (% energy) | Score 0 - Consumption≥40%  Score 1 - Otherwise |

Abbreviations: DI-GM, dietary index for gut microbiota; NHANES, National Health and Nutrition Examination Survey.

Reference:

1. Zhang X, Yang Q, Huang J, Lin H, Luo N, Tang H. Association of the newly proposed dietary index for gut microbiota and depression: the mediation effect of phenotypic age and body mass index. Eur Arch Psychiatry Clin Neurosci. Published online October 8, 2024. doi:10.1007/s00406-024-01912-x

| **Table S2 Sample characteristics, by the DI-GM group, NHANES 2007-2020(n=17,373)** | | | | | | |
| --- | --- | --- | --- | --- | --- | --- |
| **Characteristics** | **Total** | **0-3** | **4** | **5** | **>=6** | ***P*-value^a^** |
| **Age** | 57.59 ± 0.17 | 56.92 ± 0.26 | 57.11 ± 0.30 | 57.57 ± 0.29 | 58.33 ± 0.24 | **< 0.001** |
| **BMI** | 29.41 ± 0.09 | 30.37 ± 0.18 | 29.81 ± 0.15 | 29.35 ± 0.14 | 28.63 ± 0.14 | **< 0.001** |
| **Beneficial to gut microbiota** | 2.25 ± 0.02 | 0.97 ± 0.03 | 1.57 ± 0.03 | 2.20 ± 0.02 | 3.47 ± 0.02 | **< 0.001** |
| **Unfavorable to gut microbiota** | 2.61 ± 0.01 | 1.58 ± 0.02 | 2.43 ± 0.03 | 2.80 ± 0.02 | 3.21 ± 0.02 | **< 0.001** |
| **SB time** | 375.40 ± 3.05 | 374.42 ± 4.71 | 372.77 ± 5.01 | 380.00 ± 5.44 | 374.38 ± 4.62 | 0.67 |
| **Sleep duration** | 7.14 ± 0.02 | 7.12 ± 0.03 | 7.09 ± 0.04 | 7.15 ± 0.03 | 7.17 ± 0.03 | 0.31 |
| **Race/ethnicity** |  |  |  |  |  | **< 0.001** |
| Non-Hispanic Black | 3769 (9.68) | 1149 (14.78) | 961 (11.23) | 827 (8.86) | 832 (6.18) |  |
| Non-Hispanic White | 7889 (73.36) | 1656 (68.96) | 1647 (69.79) | 1872 (74.57) | 2714 (77.42) |  |
| Mexican American | 2307 (5.94) | 484 (5.70) | 613 (7.30) | 576 (6.32) | 634 (4.97) |  |
| Other races | 3408 (11.02) | 721 (10.56) | 792 (11.68) | 803 (10.25) | 1092 (11.43) |  |
| **Sex** |  |  |  |  |  | **< 0.001** |
| Female | 8774 (52.57) | 1913 (49.80) | 1992 (49.71) | 2046 (53.24) | 2823 (55.56) |  |
| Male | 8599 (47.43) | 2097 (50.20) | 2021 (50.29) | 2032 (46.76) | 2449 (44.44) |  |
| **Education level** |  |  |  |  |  | **< 0.001** |
| < high school | 4218 (15.10) | 1087 (18.79) | 1169 (18.55) | 968 (14.60) | 994 (11.04) |  |
| High school | 3970 (23.41) | 1136 (30.09) | 897 (24.23) | 930 (23.40) | 1007 (18.82) |  |
| College or above | 9185 (61.50) | 1787 (51.11) | 1947 (57.23) | 2180 (62.00) | 3271 (70.14) |  |
| **Marital level** |  |  |  |  |  | **< 0.001** |
| Married/Living with partner | 10882 (67.99) | 2398 (64.25) | 2433 (66.60) | 2626 (69.33) | 3425 (70.20) |  |
| Widowed/Divorced/Separated | 5051 (24.92) | 1204 (26.73) | 1245 (26.94) | 1130 (23.52) | 1472 (23.54) |  |
| Never married | 1440 (7.09) | 408 (9.02) | 335 (6.46) | 322 (7.15) | 375 (6.26) |  |
| **PIR** |  |  |  |  |  | **< 0.001** |
| < 1.3 | 4836 (17.29) | 1301 (22.12) | 1287 (20.35) | 1100 (16.66) | 1148 (12.90) |  |
| > 3.5 | 5985 (49.10) | 1082 (40.77) | 1180 (43.81) | 1470 (50.59) | 2253 (56.42) |  |
| 1.3-3.5 | 6552 (33.60) | 1627 (37.11) | 1546 (35.83) | 1508 (32.76) | 1871 (30.68) |  |
| **Smoking status** |  |  |  |  |  | **< 0.001** |
| Never | 8979 (51.84) | 1964 (50.95) | 2041 (51.14) | 2123 (52.21) | 2851 (52.55) |  |
| Former | 5212 (30.61) | 1148 (28.36) | 1192 (29.88) | 1179 (29.29) | 1693 (33.38) |  |
| Now | 3182 (17.55) | 898 (20.68) | 780 (18.97) | 776 (18.50) | 728 (14.08) |  |
| **Alcohol consumption** |  |  |  |  |  | **< 0.001** |
| Former | 3174 (15.86) | 808 (17.85) | 745 (16.55) | 719 (15.23) | 902 (14.67) |  |
| Heavy | 2591 (15.35) | 717 (19.61) | 662 (17.24) | 581 (14.41) | 631 (12.23) |  |
| Never | 2408 (10.33) | 503 (9.96) | 610 (11.56) | 584 (11.00) | 711 (9.33) |  |
| Moderate | 2515 (16.95) | 570 (15.94) | 611 (17.06) | 585 (17.15) | 749 (17.36) |  |
| Mild | 6685 (41.50) | 1412 (36.63) | 1385 (37.59) | 1609 (42.20) | 2279 (46.42) |  |
| **CVD** |  |  |  |  |  | **< 0.05** |
| No | 14754 (87.62) | 3351 (86.06) | 3366 (87.42) | 3473 (87.58) | 4564 (88.73) |  |
| Yes | 2619 (12.38) | 659 (13.94) | 647 (12.58) | 605 (12.42) | 708 (11.27) |  |
| **DM** |  |  |  |  |  | **< 0.001** |
| DM | 4450 (19.86) | 1175 (24.29) | 1102 (21.47) | 989 (18.86) | 1184 (16.84) |  |
| IFG | 997 (5.52) | 221 (5.57) | 258 (6.81) | 231 (4.89) | 287 (5.13) |  |
| IGT | 713 (3.87) | 150 (3.50) | 171 (4.53) | 177 (4.04) | 215 (3.56) |  |
| No | 11213 (70.75) | 2464 (66.63) | 2482 (67.19) | 2681 (72.21) | 3586 (74.46) |  |
| **Hypertension** |  |  |  |  |  | **< 0.001** |
| No | 7660 (49.68) | 1616 (45.19) | 1723 (47.92) | 1830 (50.34) | 2491 (53.06) |  |
| Yes | 9713 (50.32) | 2394 (54.81) | 2290 (52.08) | 2248 (49.66) | 2781 (46.94) |  |
| **Stroke** |  |  |  |  |  | **0.01** |
| No | 16427 (95.77) | 3759 (94.62) | 3764 (95.39) | 3876 (95.96) | 5028 (96.56) |  |
| Yes | 946 (4.23) | 251 (5.38) | 249 (4.61) | 202 (4.04) | 244 (3.44) |  |
| **PD** |  |  |  |  |  | **< 0.05** |
| No | 17141 (98.71) | 3938 (97.99) | 3965 (98.89) | 4027 (98.70) | 5211 (99.03) |  |
| Yes | 232 (1.29) | 72 (2.01) | 48 (1.11) | 51 (1.30) | 61 (0.97) |  |

Abbreviations: BMI, Body mass index; CVD, Cardiovascular disease; DM, Diabetes mellitus; DI-GM, Dietary index for gut microbiota; SB, Sedentary behavior; NHANES, National Health and Nutrition Examination Survey; PIR, Poverty income ratio; SE, Standard error; PD, Parkinson’s Disease; IFG, Impaired fasting glucose; IGT, Impaired glucose tolerance.

| **Table S3 Sample characteristics, by the Sex, NHANES 2007-2020(n=17,373)** | | | | |
| --- | --- | --- | --- | --- |
| **Characteristics** | **Total** | **Female** | **Male** | ***P*-value** |
| **Age** | 57.59 ± 0.17 | 57.90 ± 0.19 | 57.25 ± 0.20 | **< 0.001** |
| **BMI** | 29.41 ± 0.09 | 29.53 ± 0.12 | 29.28 ± 0.10 | 0.06 |
| **Beneficial to gut microbiota** | 2.25 ± 0.02 | 2.25 ± 0.03 | 2.24 ± 0.03 | 0.55 |
| **Unfavorable to gut microbiota** | 2.61 ± 0.01 | 2.69 ± 0.02 | 2.52 ± 0.02 | **< 0.001** |
| **SB time** | 375.40 ± 3.05 | 368.61 ± 3.77 | 382.92 ± 3.93 | **< 0.01** |
| **Sleep duration** | 7.14 ± 0.02 | 7.23 ± 0.03 | 7.04 ± 0.02 | **< 0.001** |
| **DI-GM** | 4.86 ± 0.03 | 4.95 ± 0.03 | 4.76 ± 0.03 | **< 0.001** |
| **Race/ethnicity** |  |  |  | 0.06 |
| Non-Hispanic Black | 3769 (9.68) | 1912 (10.14) | 1857 (9.16) |  |
| Non-Hispanic White | 7889 (73.36) | 3944 (73.15) | 3945 (73.59) |  |
| Mexican American | 2307 (5.94) | 1167 (5.74) | 1140 (6.17) |  |
| Other races | 3408 (11.02) | 1751 (10.97) | 1657 (11.07) |  |
| **DI-GM group** |  |  |  | **< 0.001** |
| 0-3 | 4010 (20.71) | 1913 (19.62) | 2097 (21.91) |  |
| 4 | 4013 (21.24) | 1992 (20.08) | 2021 (22.52) |  |
| 5 | 4078 (24.03) | 2046 (24.34) | 2032 (23.69) |  |
| >=6 | 5272 (34.02) | 2823 (35.96) | 2449 (31.88) |  |
| **Education level** |  |  |  | 0.44 |
| < high school | 4218 (15.10) | 2046 (14.80) | 2172 (15.42) |  |
| High school | 3970 (23.41) | 1977 (23.18) | 1993 (23.66) |  |
| College or above | 9185 (61.50) | 4751 (62.02) | 4434 (60.92) |  |
| **Marital level** |  |  |  | **< 0.001** |
| Married/Living with partner | 10882 (67.99) | 4763 (62.23) | 6119 (74.39) |  |
| Widowed/Divorced/Separated | 5051 (24.92) | 3249 (31.29) | 1802 (17.85) |  |
| Never married | 1440 (7.09) | 762 (6.48) | 678 (7.76) |  |
| **PIR** |  |  |  | **< 0.001** |
| < 1.3 | 4836 (17.29) | 2580 (18.45) | 2256 (16.01) |  |
| > 3.5 | 5985 (49.10) | 2863 (47.01) | 3122 (51.42) |  |
| 1.3-3.5 | 6552 (33.60) | 3331 (34.53) | 3221 (32.57) |  |
| **Smoke status** |  |  |  | **< 0.001** |
| Never | 8979 (51.84) | 5366 (57.93) | 3613 (45.09) |  |
| Former | 5212 (30.61) | 2048 (25.68) | 3164 (36.08) |  |
| Now | 3182 (17.55) | 1360 (16.39) | 1822 (18.84) |  |
| **Alcohol consumption** |  |  |  | **< 0.001** |
| Former | 3174 (15.86) | 1524 (15.66) | 1650 (16.09) |  |
| Heavy | 2591 (15.35) | 994 (12.25) | 1597 (18.78) |  |
| Never | 2408 (10.33) | 1797 (14.41) | 611 (5.82) |  |
| Moderate | 2515 (16.95) | 1583 (21.09) | 932 (12.37) |  |
| Mild | 6685 (41.50) | 2876 (36.60) | 3809 (46.93) |  |
| **CVD** |  |  |  | **< 0.001** |
| No | 14754 (87.62) | 7710 (89.62) | 7044 (85.42) |  |
| Yes | 2619 (12.38) | 1064 (10.38) | 1555 (14.58) |  |
| **DM** |  |  |  | **< 0.001** |
| DM | 4450 (19.86) | 2103 (18.25) | 2347 (21.63) |  |
| IFG | 997 (5.52) | 382 (4.10) | 615 (7.09) |  |
| IGT | 713 (3.87) | 402 (4.28) | 311 (3.42) |  |
| No | 11213 (70.75) | 5887 (73.37) | 5326 (67.86) |  |
| **Hypertension** |  |  |  | **< 0.05** |
| No | 7660 (49.68) | 3878 (50.68) | 3782 (48.58) |  |
| Yes | 9713 (50.32) | 4896 (49.32) | 4817 (51.42) |  |
| **Stroke** |  |  |  | 0.66 |
| No | 16427 (95.77) | 8312 (95.69) | 8115 (95.85) |  |
| Yes | 946 (4.23) | 462 (4.31) | 484 (4.15) |  |
| **PD** |  |  |  | **< 0.05** |
| No | 17141 (98.71) | 8647 (98.47) | 8494 (98.97) |  |
| Yes | 232 (1.29) | 127 (1.53) | 105 (1.03) |  |

Abbreviations: BMI, Body mass index; CVD, Cardiovascular disease; DM, Diabetes mellitus; DI-GM, Dietary index for gut microbiota; SB, Sedentary behavior; NHANES, National Health and Nutrition Examination Survey; PIR, Poverty income ratio; SE, Standard error; PD, Parkinson’s Disease; IFG, Impaired fasting glucose; IGT, Impaired glucose tolerance.

| **Table S4 Sample characteristics, by the age, NHANES 2007-2020(n=17,373)** | | | | |
| --- | --- | --- | --- | --- |
| **Characteristics** | **Total** | **40-59** | **60-** | ***P*-value** |
| **BMI** | 29.41 ± 0.09 | 29.54 ± 0.12 | 29.23 ± 0.12 | **0.05** |
| **Beneficial to gut microbiota** | 2.25 ± 0.02 | 2.25 ± 0.03 | 2.24 ± 0.03 | 0.88 |
| **Unfavorable to gut microbiota** | 2.61 ± 0.01 | 2.55 ± 0.02 | 2.70 ± 0.02 | **< 0.001** |
| **SB time** | 375.40 ± 3.05 | 375.85 ± 3.97 | 374.73 ± 3.32 | 0.8 |
| **Sleep duration** | 7.14 ± 0.02 | 6.98 ± 0.02 | 7.37 ± 0.02 | **< 0.001** |
| **DI-GM** | 4.86 ± 0.03 | 4.80 ± 0.03 | 4.94 ± 0.03 | **< 0.001** |
| **Sex** |  |  |  | **< 0.05** |
| Female | 8774 (52.57) | 4571 (51.70) | 4203 (53.86) |  |
| Male | 8599 (47.43) | 4300 (48.30) | 4299 (46.14) |  |
| **Race/ethnicity** |  |  |  | **< 0.001** |
| Non-Hispanic Black | 3769 (9.68) | 1981 (10.70) | 1788 (8.16) |  |
| Non-Hispanic White | 7889 (73.36) | 3613 (69.43) | 4276 (79.22) |  |
| Mexican American | 2307 (5.94) | 1336 (7.33) | 971 (3.88) |  |
| Other races | 3408 (11.02) | 1941 (12.55) | 1467 (8.74) |  |
| **DI-GM group** |  |  |  | **< 0.05** |
| 0-3 | 4010 (20.71) | 2169 (21.46) | 1841 (19.59) |  |
| 4 | 4013 (21.24) | 2101 (21.97) | 1912 (20.15) |  |
| 5 | 4078 (24.03) | 2063 (23.78) | 2015 (24.41) |  |
| >=6 | 5272 (34.02) | 2538 (32.79) | 2734 (35.85) |  |
| **Education level** |  |  |  | **< 0.001** |
| < high school | 4218 (15.10) | 1906 (14.06) | 2312 (16.63) |  |
| High school | 3970 (23.41) | 1955 (22.88) | 2015 (24.19) |  |
| College or above | 9185 (61.50) | 5010 (63.06) | 4175 (59.18) |  |
| **Marital level** |  |  |  | **< 0.001** |
| Married/Living with partner | 10882 (67.99) | 5858 (69.64) | 5024 (65.55) |  |
| Widowed/Divorced/Separated | 5051 (24.92) | 2007 (21.12) | 3044 (30.57) |  |
| Never married | 1440 (7.09) | 1006 (9.24) | 434 (3.89) |  |
| PIR |  |  |  | **< 0.001** |
| < 1.3 | 4836 (17.29) | 2484 (17.88) | 2352 (16.43) |  |
| > 3.5 | 5985 (49.10) | 3331 (52.06) | 2654 (44.70) |  |
| 1.3-3.5 | 6552 (33.60) | 3056 (30.06) | 3496 (38.88) |  |
| **Smoke status** |  |  |  | **< 0.001** |
| Never | 8979 (51.84) | 4846 (53.39) | 4133 (49.53) |  |
| Former | 5212 (30.61) | 1910 (24.48) | 3302 (39.74) |  |
| Now | 3182 (17.55) | 2115 (22.13) | 1067 (10.73) |  |
| **Alcohol consumption** |  |  |  | **< 0.001** |
| Former | 3174 (15.86) | 1238 (13.01) | 1936 (20.11) |  |
| Heavy | 2591 (15.35) | 1901 (21.23) | 690 (6.59) |  |
| Never | 2408 (10.33) | 1016 (8.26) | 1392 (13.43) |  |
| Moderate | 2515 (16.95) | 1583 (20.10) | 932 (12.26) |  |
| Mild | 6685 (41.50) | 3133 (37.40) | 3552 (47.61) |  |
| **CVD** |  |  |  | **< 0.001** |
| No | 14754 (87.62) | 8226 (93.91) | 6528 (78.26) |  |
| Yes | 2619 (12.38) | 645 (6.09) | 1974 (21.74) |  |
| **DM** |  |  |  | **< 0.001** |
| DM | 4450 (19.86) | 1642 (14.58) | 2808 (27.71) |  |
| IFG | 997 (5.52) | 473 (5.01) | 524 (6.28) |  |
| IGT | 713 (3.87) | 315 (3.28) | 398 (4.75) |  |
| No | 11213 (70.75) | 6441 (77.13) | 4772 (61.26) |  |
| **Hypertension** |  |  |  | **< 0.001** |
| No | 7660 (49.68) | 5154 (60.87) | 2506 (33.04) |  |
| Yes | 9713 (50.32) | 3717 (39.13) | 5996 (66.96) |  |
| **Stroke** |  |  |  | **< 0.001** |
| No | 16427 (95.77) | 8616 (97.66) | 7811 (92.94) |  |
| Yes | 946 (4.23) | 255 (2.34) | 691 (7.06) |  |
| **PD** |  |  |  | 0.39 |
| No | 17141 (98.71) | 8773 (98.79) | 8368 (98.58) |  |
| Yes | 232 (1.29) | 98 (1.21) | 134 (1.42) |  |

Abbreviations: BMI, Body mass index; CVD, Cardiovascular disease; DM, Diabetes mellitus; DI-GM, Dietary index for gut microbiota; SB, Sedentary behavior; NHANES, National Health and Nutrition Examination Survey; PIR, Poverty income ratio; SE, Standard error; PD, Parkinson’s Disease; IFG, Impaired fasting glucose; IGT, Impaired glucose tolerance.

| **Table S5 Sensitivity analysis of the association of DI-GM with PD, NHANES 2007-2020 (n = 17,373)** | | | | | | | | |
| --- | --- | --- | --- | --- | --- | --- | --- | --- |
|  | Crude Model | | Model 1 | | Model 2 | | Model 3 | |
| Variable | OR (95%CI) | *P*-value | OR (95%CI) | *P*-value | OR (95%CI) | *P*-value | OR (95%CI) | *P*-value |
| DI-GM | 0.843 (0.760,0.935) | 0.001 | 0.857 (0.768,0.956) | 0.006 | 0.855 (0.766,0.954) | 0.006 | 0.859 (0.769,0.959) | 0.007 |

Footnotes:

Model 1: DI_GM, Age, Sex, Race, PIR, education level, marital status, PIR, BMI, smoking status, consumption, SB time, sleep duration, DM.

Model 2: DI_GM, Age, Sex, Race, PIR, education level, marital status, PIR, BMI, smoking status, consumption, SB time, sleep duration, CVD

Model 3: DI_GM, Age, Sex, Race, PIR, education level, marital status, PIR, BMI, smoking status, consumption, SB time, sleep duration, Hypertension

Abbreviations: CI, Confidence interval; DI-GM, Dietary index for gut microbiota; NHANES, National Health and Nutrition Examination Survey; PIR, Poverty income ratio; SB, Sedentary behavior; BMI, body mass index; PD, Parkinson’s disease; DM, Diabetes mellitus; CVD, Cardiovascular disease.
